# Correlation between Neutrophil-to-Lymphocyte Ratio and Diabetic Neuropathy in Chinese Adults with Type 2 Diabetes Mellitus Using Machine Learning Methods

**DOI:** 10.1155/2024/7044644

**Published:** 2024-07-31

**Authors:** Lijie Zhu, Yang Liu, Bingyan Zheng, Danmeng Dong, Xiaoyun Xie, Liumei Hu

**Affiliations:** ^1^ Department of Interventional and Vascular Surgery Shanghai Tenth People's Hospital Tongji University School of Medicine, Shanghai, China; ^2^ Department of Geriatrics Shanghai Tongji Hospital Tongji University School of Medicine, Shanghai, China; ^3^ School of Mathematical Sciences Shanghai Jiao Tong University, Shanghai, China; ^4^ Medical School of Anhui University of Science and Technology, Huainan, Anhui 232001, China; ^5^ Department of Ophthalmology Shanghai Tenth People's Hospital Tongji University School of Medicine, Shanghai, China

## Abstract

**Objective:**

One of the most frequent consequences of diabetes mellitus has been identified as diabetic peripheral neuropathy (DPN), and numerous inflammatory disorders, including diabetes, have been documented to be reflected by the neutrophil-to-lymphocyte ratio (NLR). This study aimed to explore the correlation between peripheral blood NLR and DPN, and to evaluate whether NLR could be utilized as a novel marker for early diagnosis of DPN among those with type 2 Diabetes Mellitus (T2DM).

**Methods:**

We reviewed the medical records of 1154 diabetic patients treated at Tongji Hospital Affiliated to Tongji University from January 2022 to March 2023. These patients did not have evidence of acute infections, chronic inflammatory status within the past three months. The information included the clinical, laboratory, and demographic characteristics of the patient. Finally, a total of 442 T2DM individuals with reliable, complete, and accessible medical records were recruited, including 216 T2DM patients without complications (DM group) and 226 T2DM patients with complications of DPN (DPN group). One-way ANOVA and multivariate logistic regression were applied to analyze data from the two groups, including peripheral blood NLR values and other biomedical indices. The cohort was divided in a 7 : 3 ratio into training and internal validation datasets following feature selection and data balancing. Based on machine learning, training was conducted using extreme gradient boosting (XGBoost) and support vector machine (SVM) methods. K-fold cross-validation was applied for model assessment, and accuracy, precision, recall, *F*1-score, and the area under the receiver operating characteristic curve (AUC) were used to validate the models' discrimination and clinical applicability. Using Shapley Additive Explanations (SHAP), the top-performing model was interpreted.

**Results:**

The values of 24-hour urine volume (24H UV), lower limb arterial plaque thickness (LLAB thickness), carotid plaque thickness (CP thickness), D-dimer and onset time were significantly higher in the DPN group compared to the DM group, whereas the values of urine creatinine (UCr), total cholesterol (TC), low-density lipoprotein (LDL), alpha-fetoprotein (AFP), fasting c-peptide (FCP), and nerve conduction velocity and wave magnitude of motor and sensory nerve shown in electromyogram (EMG) were considerably lower than those in the DM group (*P* < 0.05, respectively). NLR values were significantly higher in the DPN group compared to the DM group (2.60 ± 4.82 versus 1.85 ± 0.98, *P* < 0.05). Multivariate logistic regression analysis revealed that NLR (*P* = 0.008, *C* = 0.003) was a risk factor for DPN. The multivariate logistic regression model scores were 0.6241 for accuracy, 0.6111 for precision, 0.6667 for recall, 0.6377 for *F*1, and 0.6379 for AUC. Machine learning methods, XGBoost and SVM, built prediction models, showing that NLR can predict the onset of DPN. XGBoost achieved an accuracy of 0.6541, a precision of 0.6316, a recall of 0.7273, a *F*1 value of 0.6761, and an AUC value of 0.690. SVM scored an accuracy of 0.5789, a precision of 0.5610, a recall of 0.6970, an *F*1 value of 0.6216, and an AUC value of 0.6170.

**Conclusions:**

Our findings demonstrated that NLR is highly correlated with DPN and is an independent risk factor for DPN. NLR might be a novel indicator for the early diagnosis of DPN. XGBoost and SVM models have great predictive performance and could be reliable tools for the early prediction of DPN in T2DM patients. This trial is registered with ChiCTR2400087019.

## 1. Introduction

Diabetes mellitus is a significant global public health issue. It affects nearly 500 million adults globally, and its prevalence is sharply rising [1]. It is a chronic metabolic condition characterized by poor homeostasis of glucose control. The main three types of diabetes are as follows: Type 1 diabetes (T1DM) results from autoimmune damage to the pancreas' insulin-secreting beta cells, while type 2 diabetes (T2DM) is caused by long-term insulin resistance induced by lifestyle factors, and gestational diabetes mellitus occurs during pregnancy [1]. Diabetic peripheral neuropathy (DPN) represents a significant secondary complication. It can manifest in both type 1 and type 2 diabetes, typically presenting as symmetric distal polyneuropathy, predominantly affecting the lower limbs but also potentially impacting the upper limbs and resulting in sensory loss. Chronic hyperglycemia leads to metabolic and microvascular alterations, resulting in DPN. DPN causes burning, tingling, chilly, or electric shock-like pain in 50% of instances, often worse at night [2]. Pregabalin, gabapentin, and amitriptyline are first-line antineuropathic drugs but only partially relieve symptoms [3]. Furthermore, it does not address the underlying pathophysiological mechanisms [2]. The etiology of DPN is complex and not fully understood [4]. Known mechanisms include hyperglycemia, leading to nerve damage and bioenergy depletion. Hyperglycemia can activate multiple cellular pathways, including the polyol pathway, hexosamine pathway, PKC pathway, and the accumulation of advanced glycosylation end products, which can activate the inflammatory response and damage the cell membrane and organelles [4–6]. Strong experimental and clinical data indicate that immune system activation contributes to both painful and painless forms of DPN [7]. These processes include the infiltration of peripheral macrophages and lymphocytes [8, 9], activation of microglia [10, 11], activation of the kynurenine pathway [12], and proinflammatory cytokine signaling [12–15]. A recently discovered inflammatory biomarker, the neutrophil-to-lymphocyte ratio (NLR), integrates leukocyte differentials into a single variable, providing a more accurate predictive value than each parameter alone [16]. This biomarker combines two aspects of the immune system: the innate immunological response, mainly due to neutrophils, and adaptive immunity, bolstered by lymphocytes [17]. It has been found to be closely associated with sespis [17], pneumonia [17], malignancy [18, 19], arterial diseases [20, 21], diabetic nephropathy, diabetic retinopathy, and diabetic microvascular complications. The NLR is also related to DPN, though further exploration is needed [22, 23]. Given the circumstances, this study evaluated the NLR difference between diabetic patients with and without neuropathy to better understand their relationship.

Recently, machine learning (ML) has garnered attention and acceptance among physicians due to advancements in statistical theory and computer technology [24]. The healthcare field has been transformed by ML, a branch of artificial intelligence, owing to its quick, accurate, precise, and reasonably priced computational conclusions [25]. ML is crucial for predicting various prevalent diseases, including kidney disease [24], T2DM [26], and cardiovascular disease in diabetic patients [27]. However, there are limited reports on diabetic neuropathy and machine learning. Additionally, our study aimed to establish and validate predictive models for DPN using extreme gradient boosting (XGBoost) and support vector machine (SVM) machine learning algorithms.

## 2. Materials and Methods

### 2.1. The General Population

**Figure 1:**
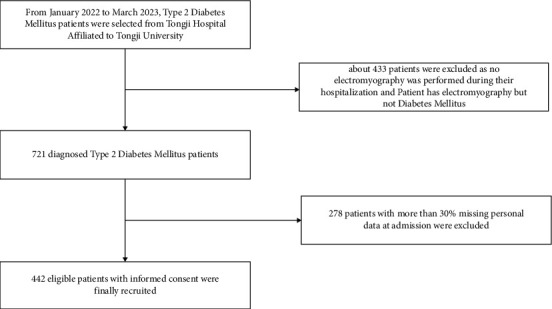
Flowchart showing the patients included in the study.

Data were collected from 1154 T2DM participants hospitalized at Tongji Hospital Affiliated to Tongji University between January 2022 and March 2023. Ultimately, 442 T2DM patients with reliable, complete, and accessible medical records were recruited, comprising 216 T2DM patients without complications (DM group) and 226 T2DM patients with DPN complications (DPN group). Enrolled patients exhibited poor blood glucose control, were managed with either oral hypoglycemic agents or insulin, and were free from recent infections. The medical information comprised 64 indicators, encompassing the patients' baseline features, blood routine examination, coagulation activity, blood biochemistry, urinary biochemistry, insulin measurement, tumor screening, ultrasound image results, and EMG results. The EMG results were used to categorize the participants into those with and without DPN.  Figure 1 shows the entire research process.

### 2.2. Gathering of Data

Patient records are screened using the corresponding criteria for inclusion and exclusion.

### 2.3. Inclusion Criteria

Diabetes was diagnosed based on the consultation guidelines of the World Health Organization as follows: fasting blood sugar (FPG) ≥ 7.0 mmol/L [126 mg/dL] and/or a 2-hour post-glucose measurement ≥ 11.1 mmol/L [200 mg/dL] [28]. After ruling out other potential causes, DPN was defined as symptoms and/or indications of nervous damage in diabetic individuals [29]. Furthermore, neuropathy was confirmed by the EMG report.

### 2.4. Exclusion Criteria

The exclusion criteria were as follows: (1) patients who left the endocrinology and metabolism department within 48 hours, (2) those aged less than 18 years or more than 89 years, (3) patients with more than 30% missing personal data at admission, and (4) smokers.

### 2.5. Research Technology

To minimize bias due to missing data, variables with more than 30% missing value were excluded from the final cohort, and other variables were imputed using the K-Nearest Neighbors (KNN) method. SPSS 26.0 software was applied to analyze the data. Normally distributed measurement data were described using the mean ± standard deviation, while non-normally distributed quantitative data were described using the median and interquartile range. One-way ANOVA and nonparametric tests were used to compare data variability between the two groups. Multivariate logistic regression was applied to analyze the relationship between different NLR levels and the occurrence of DPN. Feature selection, data preparation, balancing, modeling, and assessment were carried out using Python.

The dataset was randomly split into a training set and a validation set in a ratio of approximately 7 : 3. The training set was used to build the predictive model, and the validation set was used to verify and evaluate its performance. Two machine learning algorithms, SVM and XGBoost, were used with Python to predict the relationship between related factors and the onset of diabetic neuropathy. After using XGBoost and SVM to build a predictive DPN pathogenesis model, we aimed to understand which features had the greatest impact on the prediction results by screening the importance of features. Permutation Importance was chosen to identify the most important features in the model, and the significance of these variables was discussed. The indicators' accuracy, precision, recall, *F*1-score, confusion matrix, and area under the receiver operating characteristic curve (AUC) during 5-fold cross-validation were used to evaluate the model's performance.

### 2.6. Ethical Considerations

Written informed consent was obtained from each participant before the trial, and the research program was approved by the ethics committee of Tongji Hospital Affiliated to Tongji University, in compliance with the Declaration of Helsinki. The study has been registered with the China Experimental Registry and the registry number is ChiCTR2400087019.

## 3. Results

### 3.1. Patient Characteristic

Finally, 442 T2DM patients with reliable, complete, and accessible medical records participated in our study, including 216 T2DM patients without complications (DM group) and 226 T2DM patients with DPN (DPN group) (Table 1). The DM group consisted of 119 males and 97 females, with an average age of 61.16 ± 11.68 years, while the DPN group included 126 males and 100 females with a median age of 63.57 ± 9.67 years. Table 1 offers a comprehensive summary of the characteristics and laboratory findings of all groups. One-way ANOVA analysis between these groups indicated that there were no statistically significant differences in urinary microalbumin (UMA), urinary protein (Pro), 24-hour urinary microalbumin (24-H UMA), 24-hour urine creatinine (24-H UCr), 24-hour urinary protein (24-H pro), 24-hour urea nitrogen (24-H urea), lower extremity arterial plaque length (LLAB length), gender, systolic blood pressure (SBP), diastolic blood pressure (DBP), albumin, direct bilirubin (DBIL), total bilirubin (TBIL), alanine aminotransferase (ALT), aspartate aminotransferase (AST), urea, creatinine (Cr), uric acid (UA), triglyceride (TG), high-density lipoprotein (HDL-C), c-reactive protein (CRP), thrombocyte (PLT), neutrophils (NEUT), lymphocytes (LYM), vitamin D, carcinoembryonic antigen (CEA), carbohydrate Antigen 153 (CA153), carbohydrate antigen 125 (CA125), carbohydrate antigen199 (CA199), carbohydrate Antigen 724 (CA724), recombinant cytokeratin 19 (CK19), neuronspecificenolase (NSE), ferritin (SF), cancer antigen 50 (CA50), cancer antigen 242 (CA242), and carotid artery plaque length (CP length) (*P* > 0.05). Of the DPN patients, 131 exhibited diabetic vascular complications (DPA), compared to 109 DM patients with DPA (*P* > 0.05). Patients in the DPN group demonstrated significantly higher average NLR values compared to those in the DM group (2.60 ± 4.82 VS 1.85 ± 0.98, *P* < 0.05) (Table 1). DPN Patients exhibited elevated levels of hemoglobin A1c (HbA1C) (9.41 ± 2.44 VS 9.45 ± 2.34, *P* > 0.05) and fasting blood glucose (FBP) (7.81 ± 3.26 VS 7.85 ± 3.38, *P* > 0.05). 24-hour urine volume (24-H UV), lower limb arterial plaque thickness (LLAB thickness), carotid plaque thickness (CP thickness), D-dimer, and onset time were significantly higher in the DPN group (*P* < 0.05). The DPN cohort exhibited significantly lower levels of urine creatinine (UCr), body mass index (BMI), total cholesterol (TC), low-density lipoprotein (LDL), alpha-fetoprotein (AFP), fasting c-peptide (FCP), motor and sensory nerve conduction velocities, and conduction wave amplitude as measured by EMG (*P* < 0.05).

### 3.2. DPN's Correlation with Additional Indices

**Figure 2:**
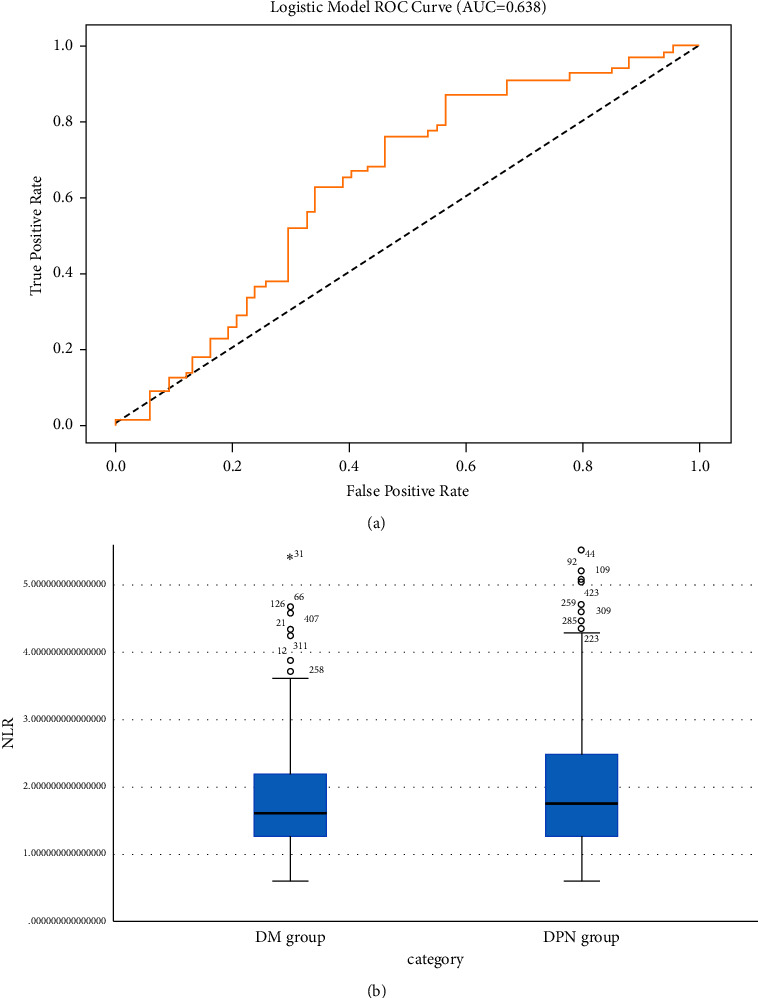
(a) ROC research for NLR to forecast diabetic peripheral neuropathy (coverage underneath curves = 0.638) and (b) average NLR results in diabetic neuropathy group and diabetic group. DM = diabetes, DPN = diabetic peripheral neuropathy, NLR = neutrophil-to-lymphocyte ratio. “∘” indicates that the individual values are more than 1.5 to 3 times the interquartile spacing (box height) from the bottom line of the box chart. “∗” indicates that the individual value is more than 3 times the box height from the top line of the box diagram.

Age, gender, body mass index, blood pressure, fasting blood sugar, serum lipid parameter, glycosylated hemoglobin, urine creatinine, uric acid, and NLR, neutrophils, and lymphocytes, among other variables, were included in the logistical regression model between the DPN group and the DM group. Diabetic neuropathy was independently correlated with UCr (*P*=0.001, *C* = −0.001), FCP (*P*=0.001, *C* = −0.003), NLR (*P*=0.008, *C* = 0.003), and LLAB thickness (*P*=0.053, *C* = 0.007), based on multivariate logistic regression findings (Table 2). The multivariate logistic regression model scores are 0.6241 for accuracy, 0.6111 for precision, 0.6667 for recall, 0.6377 for *F*1, and 0.6379 for AUC (Figure 2).

### 3.3. Model Establishment and Evaluation

NLR related DPN prediction models were developed utilizing XGBoost and SVM. The optimal parameter modeling was determined through grid search and cross-validation methods, followed by screening the importance of features using Permutation Importance.

**Figure 3:**
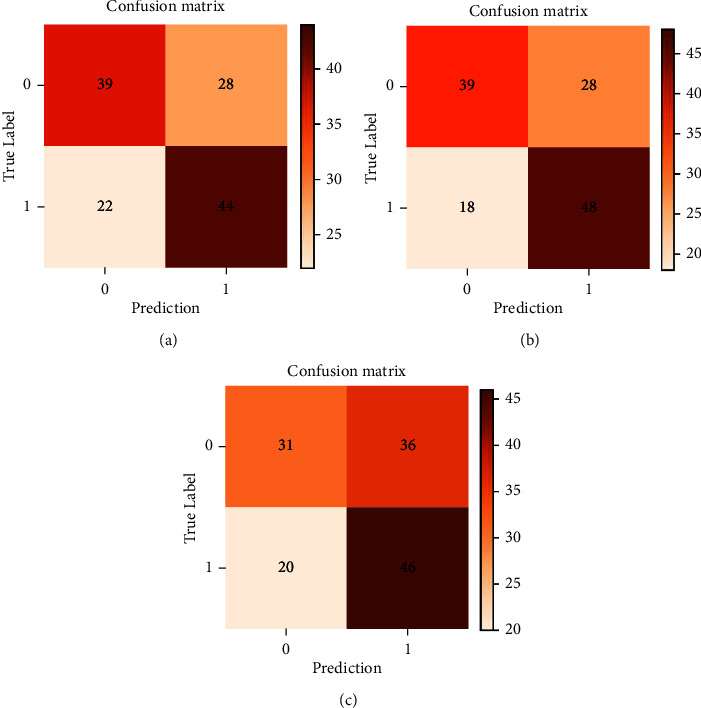
Confusion matrix of the risk prediction models with machine learning algorithms. (i) True positives (TP): cases we predicted positive and which are really positive. (ii) True negatives (TN): cases we predicted negative and which are really negative. (iii) False positives (FP): cases we predicted positive, but they are actually negative. (iv) False negatives (FN): cases we predicted negative, but they are actually positive. (a) Multivariable logistic regression analysis, (b) extreme gradient boosting (XGBoost), and (c) support vector machine (SVM).

**Figure 4:**
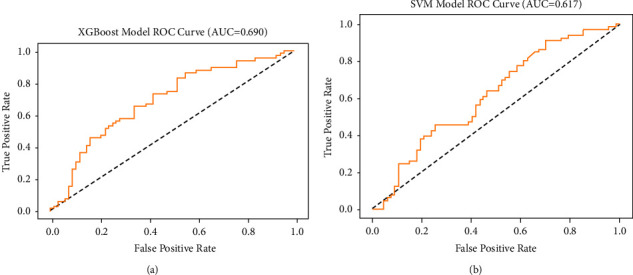
ROC curves for predicting relationship between diabetic neuropathy and the development of NLR with machine learning algorithms. (a) Extreme gradient boosting (XGBoost) and (b) support vector machine (SVM).

Additionally, the associated confusion matrix was presented in Figure 3. The metrics of the confusion matrix are denoted by true positive, true negative, false positive, and false negative. 0 represents diabetic patients, 1 represents diabetic neuropathy patients. XGBoost achieved an accuracy of 0.6541, a precision of 0.6316, a recall of 0.7273, an *F*1 value of 0.6761, and an AUC value of 0.6900. SVM achieved an accuracy of 0.5789, a precision of 0.5610, a recall of 0.6970, an *F*1 value of 0.6216, and an AUC value of 0.6170 (Figure 4), which indicated that both XGBoost and SVM had great performance in predicting the relationship between NLR and diabetic neuropathy.

### 3.4. The Importance of Characteristic Variables

**Figure 5:**
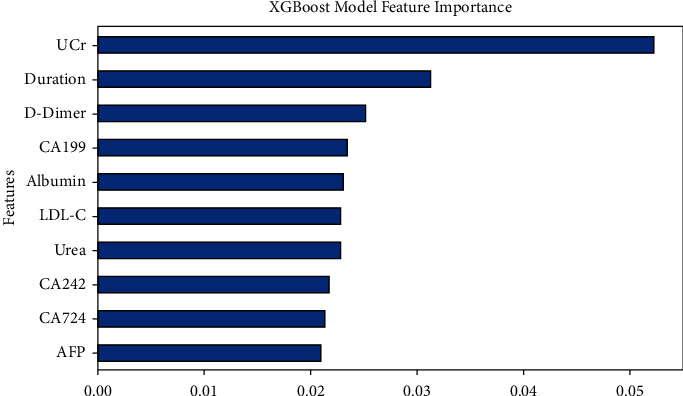
Feature significance ranking of the incorporated feature of the XGBoost model.

Based on Permutation Importance, we found that in the XGBoost machine learning approach, UCr, a measure of renal function, was the feature with the highest mean score (1^st^). This was followed by disease duration (2^nd^). D-dimer (3^rd^) is an indicator of the presence of hypercoagulability and secondary hyperfibrinolysis. CA199 (4^th^) is a common marker of gastrointestinal tumors, and can also be used to monitor the therapeutic effect and recurrence of malignant tumors. Albumin (5^th^) is the most important protein in human plasma, maintaining the body's nutrition and osmotic pressure (Figure 5).

**Figure 6:**
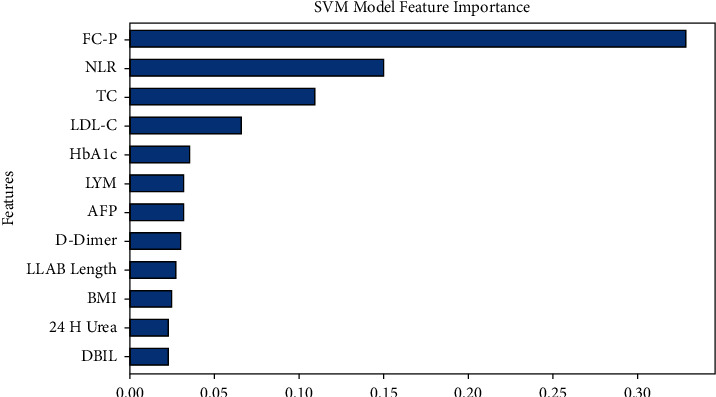
Feature significance ranking of the incorporated feature of the SVM model.

In SVM machine learning methods (Figure 6), the most important feature is FCP (1^st^), which is secreted by islet beta cells and shares a precursor with insulin. NLR (2^nd^) is a combination of the two main components of the chronic inflammatory state (neutrophils and lymphocytes). Neutrophil levels are a marker of nonspecific inflammatory processes, and lymphocyte count indicates the function of immune regulation [30]. Patients with DPN often have high neutrophils and low lymphocytes, and the NLR ratio is elevated, representing the ongoing nonspecific inflammatory state in the body and the relative deficiency in immune function. Additionally, NLR is more stable and less susceptible to interference by related factors than other leukocyte measures, including neutrophil, lymphocyte, and leukocyte counts. On the other hand, NLR has been shown to be an independent risk factor for a pathophysiological process associated with DPN called diabetic microangiopathy [28, 29, 31], which affects neurons and Schwann cells, causing neurodegeneration and leading to diabetic peripheral neuropathy. TC (3^rd^) and LDL (4^th^) are indicators of lipid metabolism, while HbA1C (5^th^) effectively reflects the average blood glucose level over the past 8 to 12 weeks.

## 4. Discussion

One of the prominent issues associated with diabetes is DPN. DPN is characterized by an insidious onset, slow progression, and initial symmetric tingling and numbness, which can progress to foot ulceration and gangrene [32]. Therefore, early diagnosis and prevention of DPN are crucial for improving the quality of life for diabetic patients.

It has been established that T2DM and its associated challenges are linked with inflammation and immunological dysfunctions [4, 33, 34]. In diabetes, chronic inflammation has been implicated in the development and progression of DPN [35, 36]. Hyperglycemia and oxidative stress are examples of stressors that might cause the production of NF-kB [36]. NF-kB activation stimulates the inflammatory response by increasing the expression of proinflammatory chemokines such as C-C motif ligand 2 (CCL2), C-X-C motif chemokine ligand 1 (CXCL1), tumor necrosis factor (TNF), and interleukins (IL-1, IL-2, IL-6, and IL-8) [5, 35, 36]. The production of IL-1 disrupts insulin signaling and leads to the degradation of insulin receptor substrate-1 (IRS-1) by neutrophils, which has been shown to contribute to insulin resistance (IR) [6]. The well-known chemotactic properties of neutrophils may exacerbate inflammation and insulin resistance in T2DM by attracting other immune cells to adipose tissue [6]. Additionally, lymphopenia may be associated with T2DM and its consequences. Similar lymphopenia has been observed in various clinical and experimental studies involving individuals with microvascular, macrovascular, and other complications [37–40]. Elevated oxidative DNA damage and the death of lymphocytes in blood vessels might contribute to this condition.

Within the present research, lymphocyte count was significantly lower in the DPN group, while neutrophil count was significantly higher. When comparing the NLR values of the DPN group to those of the DM group, they were noticeably higher (2.60 ± 4.82 vs. 1.85 ± 0.98, *P* < 0.05). Multivariate logistic regression analysis (*P*=0.008, *C* = 0.003) demonstrated that NLR is an independent risk factor for DPN. These findings indicate varying degrees of inflammation between T2DM patients with and without DPN. Given that NLR is a marker of inflammation, it might be assumed that DPN could be prevented or ameliorated by appropriately controlling chronic inflammation. Therefore, NLR might be a valuable biomarker for monitoring anti-inflammatory therapy. In addition, machine learning methods XGBoost and SVM were used to build a DPN pathogenesis model, which has been rarely reported in previous studies. This confirmed that many factors could be used to predict the occurrence of DPN. These factors included UCr, duration, D-dimer, CA199, albumin, FCP, NLR, TC, LDL-C, and HbA1C.

In machine learning models for predicting the onset of diabetic neuropathy, feature importance analysis of XGBoost and SVM showed that the top five variables of XGBoost were UCr, disease duration, D-dimer, CA199, and albumin. The top five variables for SVM were FCP, NLR, TC, LDL-C, and HbA1C. Due to differences in model prediction performance and algorithm, NLR is not always the most crucial variable. Combining the results of the two models, we conclude that controlling inflammation is important in DPN patients. UCr can be used for early diagnosis of diabetic neuropathy and kidney disease [41]. In addition, the duration of diabetes and glycosylated hemoglobin levels affect the progression of diabetic neuropathy. The longer the duration of diabetes, the higher the glycosylated hemoglobin level, and the greater the risk of complications from diabetic neuropathy [42]. Adequate nutrition is essential for tissue remodeling, and low serum albumin is a marker of malnutrition [43]. In recent years, lipid abnormalities and DPN have received increasing attention [4]. Abnormal lipid metabolism leads to atherosclerosis, and NLR is also an independent predictor of the presence of carotid plaques [44]. This should be further studied in future research. This study also found that FCP, D-dimer, and CA199 are associated with DPN and can predict its development. Fasting insulin and fasting C-peptide represent islet function, with fasting C-peptide being more representative. Higher fasting C-peptide levels were inversely correlated with DPN in this research, which aligns with a prior study that found a negative correlation between C-peptide levels and cardiovascular autonomic neuropathy in T2DM patients [45]. A previous study in Korea concluded that the risk of diabetic neuropathy was related to the lower fasting serum C-peptide quartile after adjusting for multiple confounding factors [46]. However, a Danish study found a correlation between DPN and C‐peptide levels ≥1550 pmol/L [47]. Scholars have also reported that C‐peptide improved neuropathy in type 1 diabetic BB/Wor‐rats. More research is necessary since type 1 and type 2 diabetes differ in the way C-peptide affects DPN [48].

In summary, combining multivariate logistic regression analysis and machine learning models, our results indicate that predicting DPN occurrence involves many factors, including UCr, duration, D-dimer, CA199, albumin, FCP, NLR, TC, LDL-C, and HbA1C. Our research shows that NLR is not only related to the occurrence of DPN but can also be used as a predictor to monitor the early occurrence of this disease.

According to prior research, the Diabetic Neuropathy Symptom Score (DNS) is an effective screening tool for diabetic neuropathy, and the Toronto Clinical Scoring System (CSS) can identify the presence and severity of diabetic peripheral sensory-motor polyneuropathy (DSP) [49]. However, these methods are easily influenced by patients' subjective perceptions, ignore the screening of asymptomatic neuropathy, and require more time, limiting their use in clinical DPN screening. In contrast, NLR can be easily computed using the neutrophil-to-lymphocyte ratio in peripheral blood, which is characterized by excellent stability, high repeatability, and low cost [50]. Therefore, NLR, as an early diagnostic indicator for DPN, with the potential for early identification of asymptomatic DPN patients and significant improvement in patient prognosis, holds substantial clinical significance.

The study has some limitations. First, no stratified research has been conducted on the association between NLR and DPN. Therefore, further studies are needed where individuals are categorized into different groups based on the severity of DPN. Second, our sample size is small and limited by region and ethnicity, which may lead to biases in these statistical results. Thus, multicenter studies are required to assess the use of NLR for DPN prediction in more detail.

## Figures and Tables

**Table 1:** Cluster features and test results.

Variable	Diabetes patients w/o DPN (DM group) (*n* = 216)	Diabetes patients w/DPN (DPN group) (*n* = 226)	*P* value
UMA (mg/L)	93.18 ± 327.15	102.10 ± 399.51	0.800
UCr (umol/L)	9302.07 ± 5815.50	6862.67 ± 4064.46	0.001^†^
Pro (mg/L)	201.94 ± 422.37	215.66 ± 543.77	0.773
24-H UMA (mg/24 h)	138.74 ± 447.17	163.35 ± 497.38	0.586
24-H UCr (mmol/24 h)	9.74 ± 4.53	9.29 ± 4.02	0.274
24-H UV (L/24 h)	1.74 ± 0.75	1.98 ± 0.85	0.002^†^
24-H Pro (mg/24 h)	229.92 ± 523.97	278.18 ± 640.5	0.391
24-H Urea (mol/24 h)	1.92 ± 18.67	0.32 ± 0.35	0.205
LLAB thickness (mm)	6.45 ± 7.42	8.78 ± 8.97	0.003^†^
LLAB length (mm)	1.37 ± 2.10	1.51 ± 1.39	0.409
Gender			
Male	119	126	.
Female	97	100	
Age (years)	61.16 ± 11.68	63.57 ± 9.67	0.018^†^
Duration (years)	9.89 ± 8.42	13.07 ± 8.33	0.001^†^
SBP (mmHg)	138.19 ± 18.02	138.67 ± 17.71	0.777
DBP (mmHg)	80.36 ± 12.03	79.04 ± 11.60	0.242
BMI (kg/m^2^)	25.29 ± 3.88	24.15 ± 3.22	0.001^†^
HbA1C (%)	9.41 ± 2.44	9.45 ± 2.34	0.853
Albumin (g/L)	38.71 ± 3.60	38.24 ± 5.61	0.301
DBIL (umol/L)	4.59 ± 2.13	4.47 ± 1.96	0.533
TBIL (umol/L)	12.86 ± 6.19	11.98 ± 5.98	0.138
ALT (U/L)	25.99 ± 20.65	22.66 ± 15.38	0.055
AST (U/L)	20.82 ± 12.24	19.26 ± 10.52	0.152
Urea (mmol/L)	5.92 ± 2.25	6.10 ± 2.33	0.413
Cr (umol/L)	74.70 ± 28.74	75.75 ± 30.10	0.710
UA (umol/L)	321.76 ± 91.85	321.98 ± 90.87	0.979
FBP (mmol/L)	7.81 ± 3.26	7.85 ± 3.38	0.883
TC (mmol/L)	4.74 ± 1.36	4.37 ± 1.19	0.003^†^
TG (mmol/L)	1.77 ± 1.53	2.64 ± 15.78	0.427
HDL-C (mmol/L)	1.05 ± 0.26	1.06 ± 0.26	0.737
LDL-C (mmol/L)	2.89 ± 0.96	2.59 ± 0.97	0.001^†^
D-Dimer (mg/L FEU)	0.36 ± 0.36	0.52 ± 1.02	0.029^†^
CRP (mg/L)	5.07 ± 10.50	8.39 ± 26.29	0.097
PLT (^*∗*^10^9^/L)	225.74 ± 64.98	215.83 ± 58.00	0.092
NEUT (^*∗*^10^9^/L)	3.56 ± 1.34	4.00 ± 3.76	0.106
LYM (^*∗*^10^9^/L)	2.12 ± 0.68	2.07 ± 2.16	0.707
NLR	1.85 ± 0.98	2.60 ± 4.82	0.026^†^
Vit D (ng/ml)	20.02 ± 8.75	19.95 ± 10.62	0.935
CEA (ng/ml)	3.20 ± 3.37	3.29 ± 4.10	0.811
AFP (ng/ml)	3.31 ± 6.91	2.33 ± 1.40	0.039^†^
CA153 (U/ml)	11.78 ± 6.05	11.14 ± 6.37	0.284
CA125 (U/ml)	13.48 ± 14.32	14.26 ± 18.34	0.521
CA199 (U/ml)	23.74 ± 57.03	28.14 ± 115.88	0.515
CA724 (U/ml)	4.43 ± 6.23	4.49 ± 5.57	0.919
CK19 (ng/ml)	3.11 ± 1.40	3.26 ± 1.53	0.307
NSE (ng/ml)	16.21 ± 4.62	16.01 ± 4.24	0.554
F-insulin (mU/L)	9.34 ± 12.17	9.92 ± 22.71	0.743
FCP (ng/ml)	1.99 ± 1.27	1.71 ± 1.19	0.020^†^
SF (ng/ml)	315.22 ± 227.96	267.2 ± 227.20	0.055
CA50 (U/ml)	15.15 ± 31.03	13.68 ± 17.23	0.556
CA242 (U/ml)	21.68 ± 17.40	8.39 ± 13.57	0.297
UN MFCV (m/s)	58.60 ± 32.02	51.17 ± 6.99	0.001^†^
UN MFCA (mv)	7.26 ± 1.51	6.12 ± 1.75	0.001^†^
MN MFCV (m/s)	54.77 ± 4.57	51.00 ± 6.21	0.001^†^
MN MFCA (mv)	6.51 ± 1.56	5.60 ± 1.79	0.001^†^
CPN MFCV (m/s)	46.71 ± 8.14	41.31 ± 7.29	0.001^†^
CPN MFCA (mv)	3.90 ± 1.63	2.72 ± 1.71	0.001^†^
UN SFCV (m/s)	60.09 ± 9.32	54.96 ± 11.15	0.001^†^
UN SFCA (uv)	7.94 ± 2.97	5.83 ± 4.14	0.001^†^
MN SFCV (m/s)	55.94 ± 12.14	49.47 ± 12.15	0.001^†^
MN SFCA (uv)	10.99 ± 5.70	7.06 ± 4.79	0.001^†^
CPN SFCV (m/s)	56.17 ± 13.30	50.08 ± 14.72	0.001^†^
CPN SFCA (uv)	10.19 ± 6.42	6.07 ± 4.43	0.001^†^
CP thickness (mm)	1.57 ± 1.44	1.84 ± 1.17	0.047^†^
CP length (mm)	6.10 ± 6.11	7.24 ± 5.90	0.081

Except as otherwise noted, all parameters are expressed as mean standard deviation. The significance level was set at *P* < 0.05. UMA = urinary microalbumin, UCr = urine creatinine, Pro = urinary protein, 24-H UMA = 24-hour urinary microalbumin, 24-H UCr = 24-hour urine creatinine, 24-H UV = 24-hour urinary volume, 24-H pro = 24-hour urinary protein, 24-H urea = 24-hour urea nitrogen, LLAB thickness = lower extremity arterial plaque thickness, LLAB length = lower extremity arterial plaque length, SBP = systolic blood pressure, DBP = diastolic blood pressure, BMI = body mass index, HbA1C = hemoglobin A1c, DBIL = direct bilirubin, TBIL = total bilirubin, ALT = alanine aminotransferase, AST = aspartate aminotransferase, Cr = creatinine, UA = uric acid, FBP = fasting blood glucose, TC = total cholesterol, TG = triglyceride, HDL-C = high-density lipoprotein, LDL-C = low-density lipoprotein, CRP = c-reactive protein, PLT = thrombocyte, NEUT = neutrophils, LYM = lymphocytes, NLR = neutrophil-to-lymphocyte ratio, CEA, AFP, CA153, CA125, CA199, CA724, CK19, NSE, CA50, CA242 = tumor markers, F-insulin = fasting insulin, FCP = fasting c-peptide, SF = ferritin, UN MFCV = ulnar nerve motor fiber conduction velocity, UN MFCA = ulnar nerve motor fiber conduction amplitude, MN MFCV = median nerve motor fiber conduction velocity, MN MFCA = median nerve motor fiber conduction amplitude, CPN MFCV = common peroneal nerve motor fiber conduction velocity, CPN MFCA = common peroneal nerve motor fiber conduction amplitude, UN SFCV = ulnar nerve sensory fiber conduction velocity, UN SFCA = ulnar nerve sensory fiber conduction amplitude, MN SFCV = median nerve sensory fiber conduction velocity, MN SFCA = median nerve sensory fiber conduction amplitude, CPN SFCV = common peroneal nerve sensory fiber conduction velocity, CPN SFCA = common peroneal nerve sensory fiber conduction amplitude, CP thickness = carotid artery plaque thickness, CP length = carotid artery plaque length. ^†^Significant difference between the two groups.

**Table 2:** Shows the results of logistic regression analysis of DPN.

Number	Variable	Coefficient	*P* value
1	UCr	−0.000134	0.000114
2	FCP	−0.003548	0.000603
3	NLR	0.003811	0.008709
4	LLAB thickness	0.007175	0.053946

Significance level was set at *P* < 0.05. UCr = urine creatinine, FCP = fasting c-peptide, NLR = neutrophil-to-lymphocyte ratio, LLAB thickness = lower extremity arterial plaque thickness.

## Data Availability

The original data are available from the corresponding author upon reasonable request.

## References

[1] Saeedi P., Petersohn I., Salpea P. (2019). Global and regional diabetes prevalence estimates for 2019 and projections for 2030 and 2045: results from the International Diabetes Federation Diabetes Atlas, 9(th) edition. *Diabetes Research and Clinical Practice*.

[2] Tesfaye S., Boulton A. J., Dickenson A. H. (2013). Mechanisms and management of diabetic painful distal symmetrical polyneuropathy. *Diabetes Care*.

[3] Finnerup N. B., Attal N., Haroutounian S. (2015). Pharmacotherapy for neuropathic pain in adults: a systematic review and meta-analysis. *The Lancet Neurology*.

[4] Eid S. A., Rumora A. E., Beirowski B. (2023). New perspectives in diabetic neuropathy. *Neuron*.

[5] Pang L., Lian X., Liu H. (2020). Understanding diabetic neuropathy: focus on oxidative stress. *Oxidative Medicine and Cellular Longevity*.

[6] Giovenzana A., Carnovale D., Phillips B., Petrelli A., Giannoukakis N. (2022). Neutrophils and their role in the aetiopathogenesis of type 1 and type 2 diabetes. *Diabetes*.

[7] Janahi N. M., Santos D., Blyth C., Bakhiet M., Ellis M. (2015). Diabetic peripheral neuropathy, is it an autoimmune disease?. *Immunology Letters*.

[8] Sun J. J., Tang L., Zhao X. P., Xu J. M., Xiao Y., Li H. (2019). Infiltration of blood-derived macrophages contributes to the development of diabetic neuropathy. *Journal of Immunology Research*.

[9] O’Brien J. A., McGuire H. M., Shinko D. (2021). T lymphocyte and monocyte subsets are dysregulated in type 1 diabetes patients with peripheral neuropathic pain. *Brain, Behavior, & Immunity-Health*.

[10] Yang C., Gao J., Wu B. (2017). Minocycline attenuates the development of diabetic neuropathy by inhibiting spinal cord Notch signaling in rat. *Biomedicine and Pharmacotherapy*.

[11] Zychowska M., Rojewska E., Kreiner G., Nalepa I., Przewlocka B., Mika J. (2013). Minocycline influences the anti-inflammatory interleukins and enhances the effectiveness of morphine under mice diabetic neuropathy. *Journal of Neuroimmunology*.

[12] Staats Pires A., Heng B., Tan V. X. (2020). Kynurenine, tetrahydrobiopterin, and cytokine inflammatory biomarkers in individuals affected by diabetic neuropathic pain. *Frontiers in Neuroscience*.

[13] Hussain G., Rizvi S. A., Singhal S., Zubair M., Ahmad J. (2013). Serum levels of TNF-*α* in peripheral neuropathy patients and its correlation with nerve conduction velocity in type 2 diabetes mellitus. *Diabetes and Metabolic Syndrome: Clinical Research Reviews*.

[14] Zhu T., Meng Q., Ji J., Lou X., Zhang L. (2015). Toll-like receptor 4 and tumor necrosis factor-alpha as diagnostic biomarkers for diabetic peripheral neuropathy. *Neuroscience Letters*.

[15] Hussain G., Rizvi S. A., Singhal S., Zubair M., Ahmad J. (2016). Serum levels of TGF-*β*1 in patients of diabetic peripheral neuropathy and its correlation with nerve conduction velocity in type 2 diabetes mellitus. *Diabetes and Metabolic Syndrome: Clinical Research Reviews*.

[16] He J., Bian X., Song C. (2022). High neutrophil to lymphocyte ratio with type 2 diabetes mellitus predicts poor prognosis in patients undergoing percutaneous coronary intervention: A large-scale cohort study. *Cardiovascular Diabetology*.

[17] Buonacera A., Stancanelli B., Colaci M., Malatino L. (2022). Neutrophil to lymphocyte ratio: An emerging marker of the relationships between the immune system and diseases. *International Journal of Molecular Sciences*.

[18] Cupp M. A., Cariolou M., Tzoulaki I., Aune D., Evangelou E., Berlanga-Taylor A. J. (2020). Neutrophil to lymphocyte ratio and cancer prognosis: An umbrella review of systematic reviews and meta-analyses of observational studies. *BMC Medicine*.

[19] Xiong S., Dong L., Cheng L. (2021). Neutrophils in cancer carcinogenesis and metastasis. *Journal of Hematology and Oncology*.

[20] Zhao Y., Zhang S., Yi Y. (2022). Neutrophil-to-lymphocyte ratio as a predictor for cardiovascular diseases: a cohort study in Tianjin, China. *Journal of Human Hypertension*.

[21] Meshaal M. S., Nagi A., Eldamaty A., Elnaggar W., Gaber M., Rizk H. (2019). Neutrophil-to-lymphocyte ratio (NLR) and platelet-to-lymphocyte ratio (PLR) as independent predictors of outcome in infective endocarditis (IE). *The Egyptian Heart Journal*.

[22] Chen M., Zhu Y., Wang J., Wang G., Wu Y. (2021). The predictive value of neutrophil-to-lymphocyte ratio and platelet-to-lymphocyte ratio levels of diabetic peripheral neuropathy. *Journal of Pain Research*.

[23] Ocak Z., Ahin E. M. (2022). Value of neutrophil/lymphocyte ratio in the diagnosis of diabetic neuropathy. *International Journal of Diabetes in Developing Countries*.

[24] Yue S., Li S., Huang X. (2022). Machine learning for the prediction of acute kidney injury in patients with sepsis. *Journal of Translational Medicine*.

[25] Jayatilake S., Ganegoda G. U. (2021). Involvement of machine learning tools in healthcare decision making. *Journal of Healthcare Engineering*.

[26] Wei H., Sun J., Shan W. (2022). Environmental chemical exposure dynamics and machine learning-based prediction of diabetes mellitus. *Science of the Total Environment*.

[27] Napoli C., Benincasa G., Schiano C., Salvatore M. (2020). Differential epigenetic factors in the prediction of cardiovascular risk in diabetic patients. *European Heart Journal-Cardiovascular Pharmacotherapy*.

[28] Li Y., Teng D., Shi X. (2020). Prevalence of diabetes recorded in mainland China using 2018 diagnostic criteria from the American Diabetes Association: National cross sectional study. *BMJ*.

[29] Garg A., Chilakamarri P., Koo B. B. (2021). Diagnostic and treatment considerations in restless legs syndrome complicated by diabetic neuropathy. *Current Diabetes Reports*.

[30] Núñez J., Núñez E., Bodí V. (2008). Usefulness of the neutrophil to lymphocyte ratio in predicting long-term mortality in ST segment elevation myocardial infarction. *The American Journal of Cardiology*.

[31] Hüsers J., Hafer G., Heggemann J., Wiemeyer S., John S. M., Hübner U. (2020). Predicting the amputation risk for patients with diabetic foot ulceration - a Bayesian decision support tool. *BMC Medical Informatics and Decision Making*.

[32] Callaghan B. C., Cheng H. T., Stables C. L., Smith A. L., Feldman E. L. (2012). Diabetic neuropathy: clinical manifestations and current treatments. *The Lancet Neurology*.

[33] Donath M. Y., Dinarello C. A., Mandrup-Poulsen T. (2019). Targeting innate immune mediators in type 1 and type 2 diabetes. *Nature Reviews Immunology*.

[34] Pérez-Morales R. E., Del Pino M. D., Valdivielso J. M., Ortiz A., Mora-Fernández C., Navarro-González J. F. (2019). Inflammation in diabetic kidney disease. *Nephron*.

[35] Dewanjee S., Das S., Das A. K. (2018). Molecular mechanism of diabetic neuropathy and its pharmacotherapeutic targets. *European Journal of Pharmacology*.

[36] Feldman E. L., Nave K. A., Jensen T. S., Bennett D. L. H. (2017). New horizons in diabetic neuropathy: mechanisms, bioenergetics, and pain. *Neuron*.

[37] Bodí V., Sanchis J., Núñez J. (2009). Post-reperfusion lymphopenia and microvascular obstruction in ST-segment elevation acute myocardial infarction. *Revista Espanola de Cardiologia*.

[38] Darcy C. J., Davis J. S., Woodberry T. (2011). An observational cohort study of the kynurenine to tryptophan ratio in sepsis: association with impaired immune and microvascular function. *PLoS One*.

[39] Wang R. T., Zhang J. R., Li Y., Liu T., Yu K. J. (2015). Neutrophil-Lymphocyte ratio is associated with arterial stiffness in diabetic retinopathy in type 2 diabetes. *Journal of Diabetes and its Complications*.

[40] Ulu S., Bucak A., Ulu M. S. (2014). Neutrophil-lymphocyte ratio as a new predictive and prognostic factor at the hearing loss of diabetic patients. *European Archives of Oto-Rhino-Laryngology*.

[41] Pi C. X., Gui T. J., He Q. D. (2022). Glomerular filtration rate, urine Albumin/creatinine ratio and current perception threshold in patients with diabetic kidney disease. *Diabetes Research and Clinical Practice*.

[42] Gibbons C. H., Freeman R. (2015). Treatment-induced neuropathy of diabetes: An acute, iatrogenic complication of diabetes. *Brain*.

[43] Wang S., Wang J., Zhu M. X., Tan Q. (2022). Machine learning for the prediction of minor amputation in University of Texas grade 3 diabetic foot ulcers. *PLoS One*.

[44] Corriere T., Di Marca S., Cataudella E. (2018). Neutrophil-to-Lymphocyte ratio is a strong predictor of atherosclerotic carotid plaques in older adults. *Nutrition, Metabolism, and Cardiovascular Diseases*.

[45] Wang W., Ji Q., Ran X. (2023). Prevalence and risk factors of diabetic peripheral neuropathy: a population-based cross-sectional study in China. *Diabetes*.

[46] Panero F., Novelli G., Zucco C. (2009). Fasting plasma C-peptide and micro-and macrovascular complications in a large clinic-based cohort of type 1 diabetic patients. *Diabetes Care*.

[47] Christensen D. H., Knudsen S. T., Gylfadottir S. S. (2020). Metabolic factors, lifestyle habits, and possible polyneuropathy in early type 2 diabetes: a nationwide study of 5,249 patients in the Danish centre for strategic research in type 2 diabetes (DD2) cohort. *Diabetes Care*.

[48] Zhang W., Kamiya H., Ekberg K., Wahren J., Sima A. A. (2007). C-peptide improves neuropathy in type 1 diabetic BB/Wor-rats. *Diabetes*.

[49] Carmichael J., Fadavi H., Ishibashi F., Shore A. C., Tavakoli M. (2021). Advances in screening, early diagnosis and accurate staging of diabetic neuropathy. *Frontiers in Endocrinology*.

[50] Xu T., Weng Z., Pei C. (2017). The relationship between neutrophil-to-lymphocyte ratio and diabetic peripheral neuropathy in Type 2 diabetes mellitus. *Medicine (Baltimore)*.

